# RNA Sequencing Revealed a Weak Response of Gingival Fibroblasts Exposed to Hyaluronic Acid

**DOI:** 10.3390/bioengineering11121307

**Published:** 2024-12-23

**Authors:** Layla Panahipour, Atefe Imani, Natália dos Santos Sanches, Hannes Kühtreiber, Michael Mildner, Reinhard Gruber

**Affiliations:** 1 Department of Oral Biology, University Clinic of Dentistry, Medical University of Vienna, Sensengasse 2a, 1090 Vienna, Austria; layla.panahipour@meduniwien.ac.at (L.P.); dr_a_imani@hotmail.com (A.I.); natalia.s.sanches@unesp.br (N.d.S.S.); 2 Department of Diagnosis and Surgery, Araçatuba Dental School of Sao Paulo, Sao Paulo 16015-050, Brazil; 3 Department of Dermatology, Medical University of Vienna, 1090 Vienna, Austria; n01471143@students.meduniwien.ac.at (H.K.); michael.mildner@meduniwien.ac.at (M.M.); 4 Department of Periodontology, School of Dental Medicine, University of Bern, 3010 Bern, Switzerland; 5 Austrian Cluster for Tissue Regeneration, 1200 Vienna, Austria

**Keywords:** hyaluronic acid, gingival fibroblasts, macrophages, bulk RNAseq, guided bone regeneration, soft tissue augmentation, gingival recessions, infrabony defects

## Abstract

Hyaluronic acid was proposed to support soft tissue recession surgery and guided tissue regeneration. The molecular mechanisms through which hyaluronic acid modulates the response of connective tissue cells remain elusive. To elucidate the impact of hyaluronic acid on the connective tissue cells, we used bulk RNA sequencing to determine the changes in the genetic signature of gingival fibroblasts exposed to 1.6% cross-linked hyaluronic acid and 0.2% natural hyaluronic acid. Transcriptome-wide changes were modest. Even when implementing a minimum of 1.5 log2 fold-change and a significance threshold of 1.0 −log10, only a dozenth of genes were differentially expressed. Upregulated genes were PLK3, SLC16A6, IL6, HBEGF, DGKE, DUSP4, PTGS2, FOXC2, ATAD2B, NFATC2, and downregulated genes were MMP24 and PLXNA2. RT-PCR analysis supported the impact of hyaluronic acid on increasing the expression of a selected gene panel. The findings from bulk RNA sequencing suggest that gingival fibroblasts experience weak changes in their transcriptome when exposed to hyaluronic acid.

## 1. Introduction

Hyaluronic acid (HAc) is a non-sulfated glycosaminoglycan composed of repeating β(1–4)-glucuronic acid and β(1ߝ3)-N-acetyl glucosamine heterodimers. HAc is a major component of the extracellular matrix predominantly produced by cells of the mesenchymal lineage, represented by synoviocytes and chondrocytes in a joint, with its main function in the synovial fluid integrating viscoelasticity and hygroscopic properties, and bolstering cartilage to become a shock absorber [1,2]. Hyaluronic acid is further present in the skin [3], eyes [4,5], and, as highlighted in this study, the soft tissue of the oral cavity [6]. Three different synthases, the isozymes HAS1, HAS2, and HAS3, all of which are membrane-spanning, are capable of producing and releasing the polymer into the extracellular matrix, are expressed in periodontal fibroblasts [7]. The synthases in interplay with endocytic hyaluronidases [8] are required for the permanent turnover of HAc.

The accessibility of HAc is not restricted to its viscoelasticity and hygroscopic properties as HAc can bind to cell surface receptors [9]: (i) the transmembrane glycoprotein CD44 [10,11], (ii) the receptor for hyaluronate-mediated motility (RHAMM) [12], and (iii) ICAM-1 [13]. However, CD44 does not exclusively bind HAc as it interacts with other ligands such as osteopontin, collagens, and matrix metalloproteinases. CD44 also mediates the uptake and degradation of hyaluronan [14]. Moreover, RNAseq screening has identified the hyaluronic acid–GPRC5C (G protein-coupled receptor class C group 5 member C) signaling axis controlling hematogenic stem cell niches [15]. In general, however, knowledge of how hyaluronic acid affects cell signaling is scare and the respective changes in the genetic signature are still emerging—also in dentistry.

The oral soft tissue has a delicate structural and immunological function that goes beyond the protection of the tooth-bearing periodontium against the microbial burden of the oral cavity and the damage caused by mastication [16,17]. The oral mucosa and the periodontal soft tissue basically consist of a fibroblast-rich connective tissue shielded towards the oral cavity by the epithelium [16]. Consistent with other barriers, such as the skin, periodontal fibroblasts produce major proteoglycans [7,18] and express CD44 in vivo [19] and in vitro [20,21,22,23,24]. Apart from the endogenously produced HAc by the gingival fibroblasts [7], clinically, hyaluronic acid became recognized for its adjunctive use in dentistry [25]. It is particular cross-linked HAc combined with natural HAc that has been studied in preclinical models [26,27,28] and tested for its clinical use in dentistry, e.g., in intrabony periodontal defects [29], to preserve the buccal tissue volume [30] and in alveolar ridge preservation [31]. Moreover, HAc was studied as an adjunctive in the treatment of gingival recessions [32] in periodontal surgery of infrabony defects [33] upon topical application to support wound healing after a palatal graft harvesting [34], and to overcome interdental papillary deficiencies [35]. Together, these clinical observations provide the scientific fundament for our in vitro study aiming to uncover the underlying molecular mechanism, thus answering the fundamental question on how hyaluronic acid affects the response of oral cells, particularly gingival fibroblasts.

Previous in vitro research was based on precoating tissue culture plates with diluted HAc containing cross-linked HAc prior to cell seeding—presumably allowing the cells to float on the viscous HAc matrix. This coating caused a decreasing osteogenic differentiation of periodontal ligament fibroblasts [36], murine mesenchymal stromal ST2 cells, and osteogenic MC3T3-E1 cells [37]. In a similar setting, cross-linked HAc and native HAc led to an increased expression of cytokines in oral fibroblasts [38]. Functional studies were also performed with respect to biofilm quantity, and the expression of IL8 and HAc receptors was reported [24]. Precoating may impact gene expression, e.g., dermal fibroblasts develop an inflammatory phenotype when they grow in relaxed collagen gels or on modified collagen sponges, compared to tissue culture plates [39,40]. Here, we propose a traditional approach that first seeds the fibroblasts onto regular tissue culture plates and, once attached, exposes the cells to cross-linked HAc and native HAc, followed by bulk RNAseq screening. We have implemented RNAseq screening to uncover the transcriptomic signature change of the fibroblasts upon exposure to HAc based on deep-sequencing technologies [41], similar to what we used to study the cell response to platelet-rich fibrin [42]. RNAseq unfolds the extent and complexity of transcriptomes, which allows the functional interpretation of the signature change.

The aim of the present research is, therefore, to take advantage of RNAseq technology to identify strongly regulated genes in gingival fibroblasts being exposed to a diluted preparation of commercially available HAc, inspired by the clinical scenario of HAc application to support periodontal regeneration.

## 2. Materials and Methods

### 2.1. Gingival Fibroblasts and Macrophages

Explant cultures of gingiva from three healthy individuals who provided informed consent were used to isolate gingival fibroblasts from extracted wisdom teeth. Approval from the Ethical Committee of the Medical University of Vienna (EK #631/2007) is available. The fibroblasts were isolated and expanded in DMEM supplemented with 10% fetal calf serum (FCS) and 1% antibiotics (Invitrogen Corporation, Carlsbad, CA, USA). A pool of three cell donors, all at low passage, were seeded at 30,000 cells/cm^2^ into 24-well plates and exposed to diluted hyaDENT BG (Regedent AG; Zurich, Switzerland), a medical device, termed HAc, throughout the manuscript. It consists of 16.0 mg/mL of cross-linked hyaluronic acid and 2.0 mg/mL of hyaluronic acid with sodium chloride. Cross-linking was performed with butanediol diglycidyl ether of 1000 kDa-HAc monomers [24,37,38]. The gingival fibroblasts were exposed to a 4.5-fold diluted hyaDENT BG, resulting in a final concentration of a total of 4 mg/mL cross-linked and native HAc for 6 h in serum-free DMEM followed by the isolation of total RNA. This preparation is termed HAc throughout the manuscript.

To generate macrophages, bone marrow cells from BALB/c mice (Animal Research Laboratories, Himberg, Austria) were seeded at 1 × 10^6^ cells/cm^2^ into 24-well plates (VWR International, Radnor, PA, USA) and expanded for 5 days in growth medium containing 20 ng/mL mouse macrophage colony-stimulating factor (M-CSF; ProSpec-Tany TechnoGene, Rehovot, Israel). Both cell types were exposed to HAc for 6 h in serum-free DMEM, followed by total RNA isolation. For gingival fibroblasts, human IL1β and TNFα (ProSpec-Tany TechnoGene Ltd., Rehovot, Israel), both at 10 ng/mL and for murine macrophages, LPS from Escherichia coli 0111:B41 (Sigma–Aldrich, St. Louis, MO, USA) at 100 ng/mL was used as a positive control to boost the expression of inflammatory genes.

### 2.2. Total RNA Isolation, Sequencing, and Data Analysis

Total RNA was isolated with the GeneMATRIX Universal RNA purification kit with DNAse digestion (EUR_X_, Gdańsk, Poland). Sequencing libraries from total RNA were prepared for the fibroblasts at the Core Facility Genomics, Medical University of Vienna, using the QuantSeq 3′ FWD protocol version 2 with unique dual indices (Lexogen GmbH, Vienna, Austria). Fifteen PCR cycles were used for library prep, as determined by qPCR according to the library prep manual. Libraries were QC-checked on a Bioanalyzer 2100 (Agilent Technologies, Santa Clara, CA, USA) using a high-sensitivity DNA Kit for correct insert size and quantified using Qubit dsDNA HS Assay (Invitrogen, Waltham, MA, USA). Pooled libraries were sequenced on a P2 flowcell on a NextSeq2000 instrument (Illumina, San Diego, CA, USA) in 1 × 75 bp single-end sequencing mode. On average, 7 million reads per sample were generated. Reads in fastq format were generated using the Illumina bcl2fastq command line tool (v2.19.1.403) and the Lexogen idemux tool for optimal demultiplexing of long unique dual indices. Reads were trimmed and filtered using cutadapt version 2.8 to trim polyA tails, remove reads with N’s, and trim bases with a quality of less than 30 from the 3′ ends of the reads [43]. On average, 5 million reads were left after this procedure. Trimmed reads in fastq format were aligned to the human reference genome version GRCh38 with Gencode 29 annotations using STAR aligner [44] version 2.6.1a in 2-pass mode. STAR counted raw reads per gene. Differential gene expression was calculated using DESeq2 [45] version 1.22.2.

### 2.3. Reverse Transcription Quantitative Real-Time PCR (RT-qPCR)

Total RNA was transcribed into cDNA (LabQ technology, Labconsulting, Vienna, Austria). Amplification was performed on a CFX Connect™ Real-Time PCR Detection System (Bio-Rad Laboratories, Hercules, CA, USA). The selection of target genes was based on RNAseq, and the primers are listed in Table 1 and Table 2. The quantification of individual mRNA levels was normalized to the expression of GAPDH using the ∆∆Ct method. Relative mRNA expression levels are normalized to the unstimulated control.

### 2.4. Volcano Plot, Heat Map, and Gene Set Enrichment Analysis

PCAGO was applied as a web-based service for principal component analysis (PCA) with a variance cut-off at 500 genes. Zero read counts were removed [46]. For volcano plot generation, we used VolcaNoseR, a web-based tool [47]. The up-and-down-regulated genes were used for further analysis under the premise of a minimum log2 1.5-fold change and a minus log10 significance level of 1.0 [47]. Heat map analysis was performed with R from genes that were significantly changed, considering an adjusted *p*-value < 0.05 (www.R-project.org) (accessed on 20 September 2024). The g:Profiler was used as a functional enrichment analysis tool that integrates many databases, including Gene Ontology [48]. The sequence-based data are presented in the Supplement Files.

**Table 1:** Human primer list.

ATAD2B-F: ATTCATGCGCTAAAGGAAATGGT	ATAD2B-R: AGGAGGGCCATAAAACAAACAG [49]
CXCL1-F: TCCTGCATCCCCCATAGTTA	CXCL1-R: CTTCAGGAACAGCCACCAGT [50]
CXCL10-F TGCCATTCTGATTTGCTGCC	CXCL10-R: TGCAGGTACAGCGTACAGTT [51]
CXCL2-F: CCCATGGTTAAGAAAATCATCG	CXCL2-R: CTTCAGGAACAGCCACCAAT [52]
DGKE-F: GACGGGCACCTGATCTTGTG	DGKE-R: CTGGAGGCTACACCAGAAGG [53]
DUSP4-F: GGGGTCCTGTGGAGATCCTT	DUSP4-R: GGCAGTCCGAGGAGACATTC [54]
FOXC2-F: CCTCCTGGTATCTCAACCACA	FOXC2-R: GAGGGTCGAGTTCTCAATCCC [55]
GAPDH-F: AGCCACATCGCTCAGACAC	GAPDH-R: GCCCAATACGACCAAATCC [56]
HBEGF-F: ATCGTGGGGCTTCTCATGTTT	HBEGF-R: TTAGTCATGCCCAACTTCACTTT [57]
IL8/CXCL8-F: AACTTCTCCACAACCCTCTG	IL8/CXCL8-R: TTGGCAGCCTTCCTGATTTC [58]
PLK3-F: TTTTCGCACCACTTTGAGGAC	PLK3-R: GAGGCCAGAAAGGATCTGCC [59]
PTGS2-F: CCTGTGCCTGATGATTGC	PTGS2-R: CTGATGCGTGAAGTGCTG [60]
SLC16A6-F: CGCTGTGTTTGCTTTCGCACCA	SLC16A6-R: TTTTCGGTGACGCTGGTCCTCT [61]

**Table 2:** Mouse primer list.

IL1-F: TTGGTTAAATGACCTGCAACA	IL1-R: GAGCGCTCACGAACAGTTG [62]
IL6-F: GCTACCAAACTGGATATAATCAGGA	IL6-R: CCAGGTAGCTATGG-TACTCCAGAA [63]
GAPDH-F: AACTTTGGCATTGTGGAAGG	GAPDH-R: GGATGCAGGGATGATGTTCT [64]

## 3. Results

### 3.1. Principal Component Analysis and Heat Map of Gene Expression Changes by HAc

**Figure 1:**
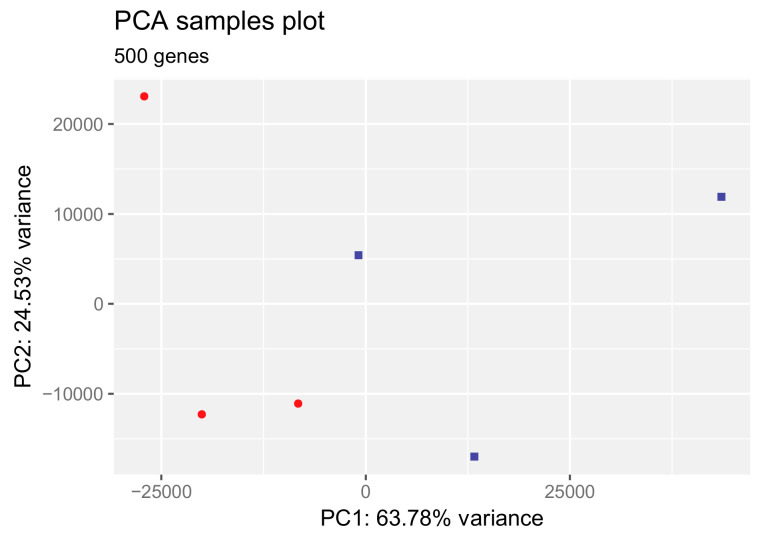
Principal component analysis for differentially expressed genes in gingival fibroblasts treated with HAc. The plot shows the projection of the top 500 genes onto the two-dimensional space spanned by the first and second principal components (PC1, PC2). The expression levels used as input are raw gene counts. Red is control, blue is HAc.

To investigate the degree to which HAc alters the transcriptional signature of human gingival fibroblasts, we conducted bulk RNA sequencing of gingival fibroblasts exposed to 4 mg/mL HAc for 6 h. The list of regulated genes is presented in the Supplement File S1. The high sensitivity of this particular preparation of gingival fibroblasts to potential agonists is supported by a sister experiment where lysates of platelet-rich fibrin caused a robust change in gene expression, including increased levels of chemokines and IL6 [42]. PCA analysis was based on the 500 most relevant genes, with PC1 and PC2 explaining 64% and 25% of the changes, respectively (Supplement File S2). The PCA was based on the raw counts of differentially expressed genes. In contrast to the sharp response to PRF [42], exposure of the fibroblasts to HAc only caused a moderate separation within the two dimensions in the PCA, thus no obvious clustering (Figure 1).

### 3.2. Volcano Blot of Gene Expression Changes by HAc

**Figure 2:**
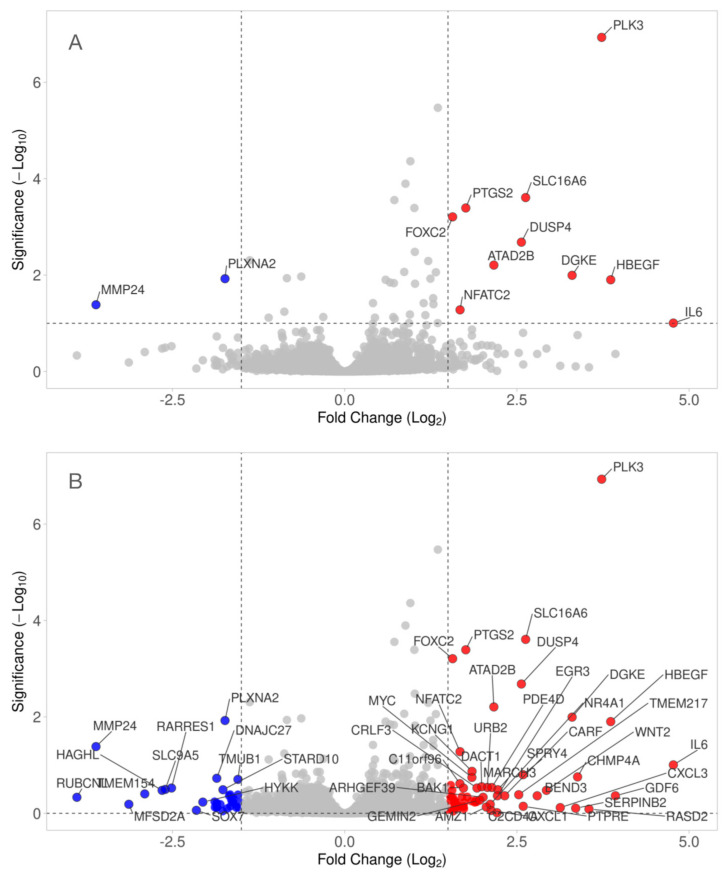
Volcano plot analysis of differentially expressed genes in gingival fibroblasts treated with HAc. Volcano plot analysis identified upregulated (red) and downregulated (blue) genes in gingival fibroblasts treated with HAc. The annotated dots are data points with the largest (Manhattan) distance from the origin and are above the thresholds indicated by the dashed line. (**A**) The threshold was set to at least a 4.0-fold change and a significance level of *p* = 0.1. (**B**) Volcano plot of the top 50 genes with no statistical threshold.

Next, we created a Volcano plot to identify changes in our data set (Supplement Files S3). Our volcano plot combines a threshold of statistical significance of 1.0 −Log10 (*p* = 0.1) with the 1.5 Log2 magnitude of the change (4-fold) to display. Based on this moderate threshold, and even though the same cells remarkably responded to PRF [42], only PLK3, SLC16A6, IL6, HBEGF, DGKE, DUSP4, PTGS2, FOXC2, ATAD2B, NFATC2 (upregulated), and MMP24 and PLXNA2 (downregulated) reached the undiscriminating level of significance together with an at least 4-fold upregulation when compared to unstimulated fibroblasts (Figure 2A). However, 98 genes appear when the significance threshold is eliminated (Figure 2B) and 22 genes are regulated based on an adjusted *p*-value of *p* = 0.05 only (Supplement File S1). 

### 3.3. RT-PCR Gene Expression Changes by HAc

**Figure 3:**
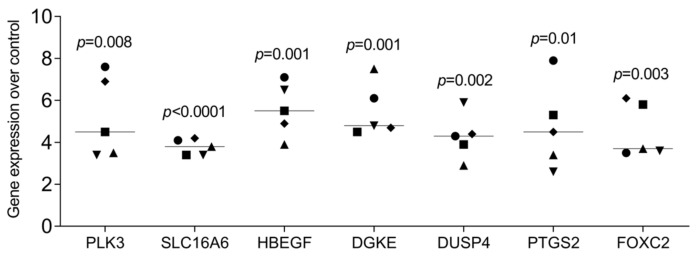
RT-PCR analysis of differentially expressed genes in gingival fibroblasts treated with HAc. Gingival fibroblasts were seeded onto a tissue culture-treated surface and, the following day, exposed to 3.6 mg/mL of HAc cross-linked and 0.4 mg/mL of HAc with sodium chloride for 6 h in serum-free DMEM followed by RT-PCR analysis. Gene expression changes were calculated by the ΔΔCT method, and findings are expressed as the x-fold increase compared to unstimulated cells, which have an expression level of 1. Statistics are based on a ratio-paired *t*-test, and data points represent independent experiments.

**Figure 4:**
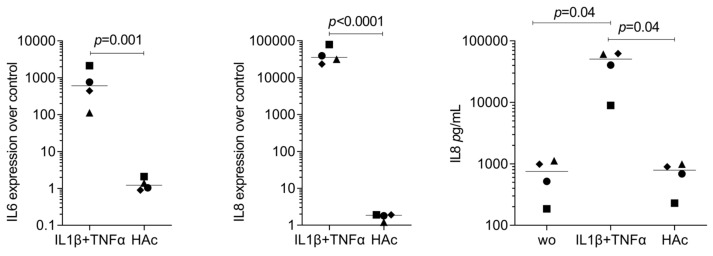
RT-PCR and ELISA analysis of inflammatory genes in gingival fibroblasts treated with HAc. Gingival fibroblasts were seeded onto a tissue culture-treated surface and, the following day, exposed to 3.6 mg/mL of HAc cross-linked and 0.4 mg/mL of HAc or IL1β and TNFα at 10 ng/mL for 6 h in serum-free DMEM followed by RT-PCR analysis. Gene expression changes were calculated by the ΔΔCT method, and findings are expressed as an x-fold increase compared to unstimulated cells, which have an expression level of 1. Statistics are based on a ratio-paired *t*-test. Data points represent independent experiments.

**Figure 5:**
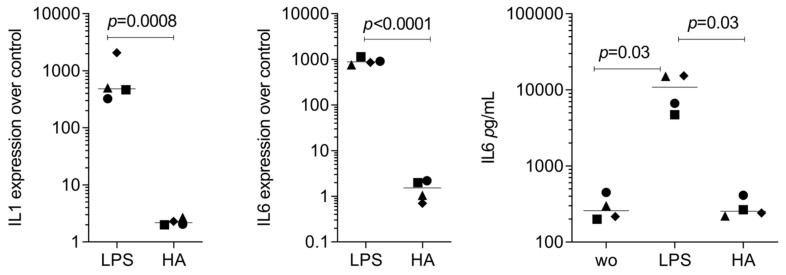
RT-PCR and ELISA analysis of inflammatory genes in primary murine macrophages treated with HAc. Murine bone marrow-derived macrophages were exposed to 3.6 mg/mL of cross-linked HAc and 0.4 mg/mL of HAc or LPS from *E. coli* at 100 ng/mL for 6 h in serum-free DMEM followed by RT-PCR analysis. Gene expression changes were calculated by the ΔΔCT method, and findings are expressed as an x-fold increase compared to unstimulated cells, which have an expression level of 1. Statistics is based on a ratio-paired *t*-test. Data points represent four independent experiments.

As a second approach, we confirmed by RT-PCR whether gingival fibroblast transcriptomes evolved with HAc exposure. Compared with untreated cells, HAc exposure causes the expected increase of a gene panel we have selected based on the Volcano plot, e.g., PLK3, SLC16A6, HBEGF, DGKE, DUSP4, PTGS2, and FOXC2 (Figure 3). Considering that PTGS2 is linked to inflammation and IL6 is an indicator cytokine, we could further show that HAc moderately enhanced the expression of inflammatory IL6 and CXCL8/IL8, but by far not reaching the high levels when gingiva fibroblasts are exposed to IL1β and TNFα (Figure 4). In addition, we have tested the impact of HAc on a potential inflammatory response in murine primary macrophages. There were no significant changes in IL1 and IL6 expression, in contrast to LPS exposure (Figure 5). Thus, HAc marginally increased inflammatory cytokines and chemokines but considerably less than with IL1β, TNFα, or PRF lysates in gingival fibroblasts [42]. HAc had no significant impact on the expression of CXCL1, CXCL2, and CXCL10 or in reducing the inflammatory response caused by IL1β, TNFα, and LPS in gingival fibroblasts and murine macrophages, respectively (Supplementary File S3).

### 3.4. Heat Map of Gene Expression Changes by HAc

**Figure 6:**
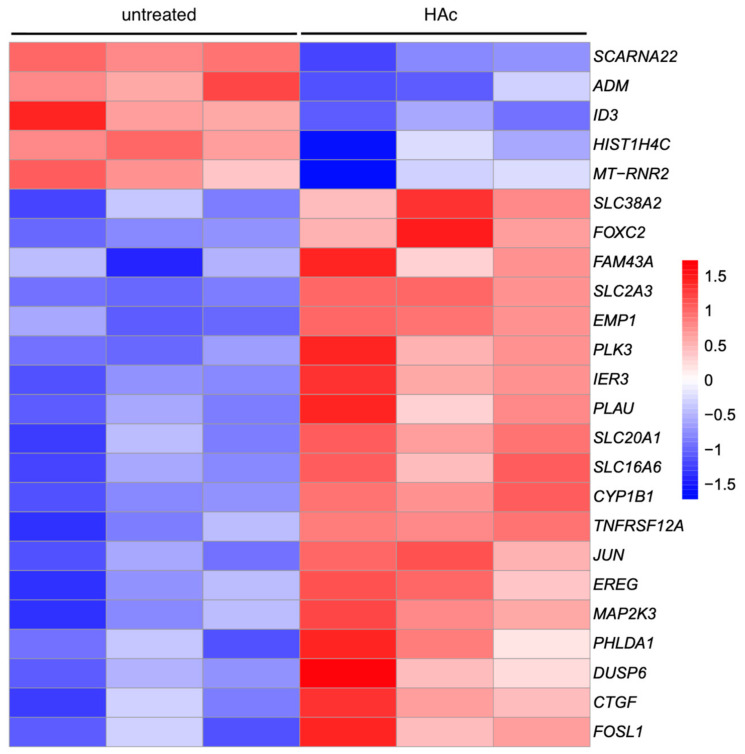
Heat map of differentially expressed genes in gingival fibroblasts treated with HAc. The heat map is used to visualize the differentially expressed genes in untreated fibroblasts versus the fibroblasts exposed to HAc. The data set used was the differentially expressed genes with an adjusted *p*-value of *p* < 0.05. This heatmap integrates the data from the three different fibroblast preparations. Turning from red to blue represents downregulated genes, and from blue to red represents upregulated genes. The color intensity reflects the expression levels of the raw data.

To be less stringent than the Volcano plot, we performed a heatmap analysis based on the significantly regulated genes with an adjusted *p*-value of *p* < 0.05 (Supplement File S4). Based on this criterion, we identified 19 and 5 as significantly up- and down-regulated genes, respectively (Figure 6). However, as expected from the Volcano plot, the overall intensity of the 24 gene expression changes was low with a 1.04 (min 0.35, max 3.73) and −0.63 (min −1.01, max −0.56) log2 fold-change. Moreover, the most strongly regulated genes that also appeared in the Volcano analysis were PLK3 (3.7 log2-fold), SLC16A6 (2.6 log2-fold), and FOXC2 (1.6 log2-fold), while other genes identified in the Volcano analysis—HBEGF, DGKE, DUSP4, PTGS2 and ATAD2B, and downregulated PLXNA2—failed to appear in the heat map. Thus, the heat map preferentially depicts genes with a homogenous distribution within the data set rather than reflecting a substantial signature change of gingival fibroblasts exposed to HAc.

### 3.5. STRING Cluster Analysis of Differentially Expressed Genes by HAc

**Figure 7:**
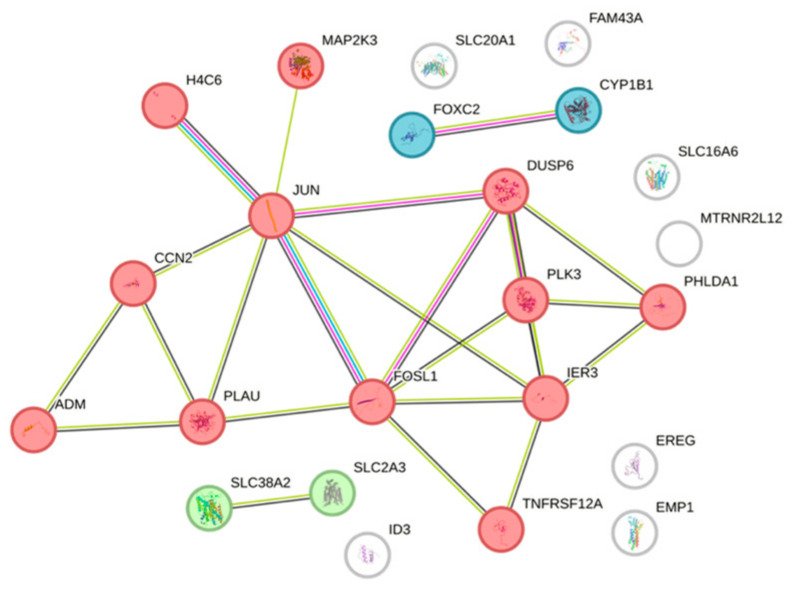
STRING analysis of expression changes by HAc. Protein–protein association network and functional enrichment analyses of the 24 genes with a differential expression in gingival fibroblasts exposed to HAc. We identified three clusters of genes.

We next investigate the clustering of the 24 genes with an adjusted *p*-value of *p* < 0.05 based on a STRING analysis (Supplement Files S5). We identified 16 genes distributed in three clusters, (red) ADM, CCN2, DUSP6, FOSL1, H4C6, IER3, JUN, MAP2K3, PHLDA1, PLAU, PLK3, TNFRSF12A, with eight genes linked to apoptosis in g:Profiler, (green) SLC2A3, SLC38A2, and (blue) CYP1B1, FOXC2 (Figure 7).

### 3.6. g:Profiler Analysis of Gene Expression Changes by HAc

**Figure 8:**
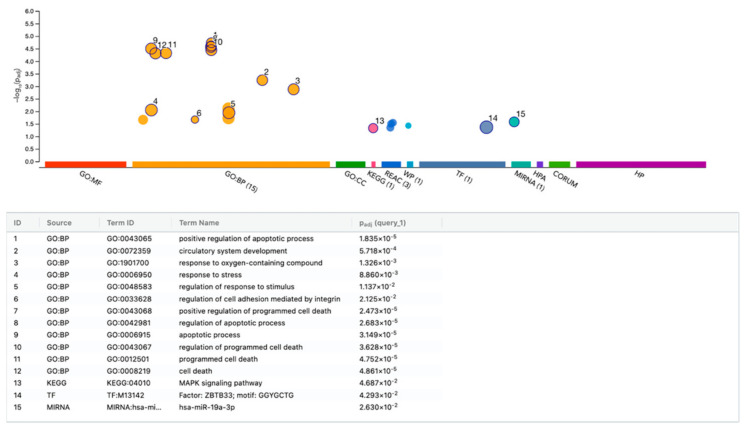
g:Profiler analysis of differentially expressed genes in gingival fibroblasts treated with HAc. Enrichment analysis was performed using the g:Profiler online tool based on the selected 24 genes. Selected top significant pathways were highlighted and labeled numerically. Using the Benjamini–Hochberg method, the *p*-value was adjusted (Padj) for multiple tests.

Finally, we performed a functional enrichment analysis of the 24 genes with an adjusted *p*-value of *p* < 0.05 in gingival fibroblasts exposed to HAc (Figure 8, Supplement Files S6). The g:Profiler analysis revealed significant enrichment of genes that are linked mainly to apoptosis, including CYP1B1, IER3, PLK3, DUSP6, ID3, PHLDA1, JUN, TNFRSF12A, FOXC2, ADM, FOSL1, EMP1—as well as transcription factor ZBTB33 [65] and miRNA miR-19a-3p [66]. These data should be viewed under the premise that g:Profiler does not consider the low magnitude of gene expression changes.

## 4. Discussion

Hyaluronic acid has become a widely used adjunctive device in dentistry [25], including the treatment of gingival recessions [32] with cross-linked HAc culminating in a series of preclinical studies [26,27,28] and clinical research [29,30,31,32,33,34,35]. This accumulating evidence has prompted basic research to understand the local response to HAc activity at a cellular level. We have acknowledged this demand to know more about how cells of the connective tissue, represented by gingival fibroblasts, respond to a clinically established device consisting of cross-linked and non-cross-linked hyaluronic acid. Our bioassay was based on a standard experimental setting where the gingival fibroblasts grown on the surface of a tissue culture plate, similar to what was recently reported in dermatology [67], were exposed to a commercially available but diluted HAc preparation. We have chosen RNAseq to uncover the differentially expressed genes and, thus, the impact of HAc on the transcriptome. Our main finding was that the overall response of gingival fibroblasts to HAc could be restricted to 12 genes with a > 4-fold change and adjusted *p* < 0.1, and 21 genes when exclusively limiting to adjusted *p* < 0.05. In essence, we report the transcriptome changes of gingival fibroblasts when exposed to a clinically approved available preparation of HAc.

Building on the findings of others, we now integrate our results to provide a more comprehensive perspective. We acknowledge work where the exact source of HAc, and even the same concentration of HAc, provoked a significant expression of inflammatory cytokines IL1β and TNFα in oral fibroblasts [67]. However, in their setting, cells were seeded onto HAc-precoated plates. In a similar setting, no changes in IL1β expression were observed with periodontal ligament fibroblasts [36], similar to what we have observed, as neither the cytokines nor chemokines reached an expression level that exceeds a relevant threshold. Consistently, HAc fragments are not inducers of inflammatory cytokine expression in synovial fibroblasts, chondrocytes, or peripheral blood mononucleated cells [68]. Thus, in our setting, where gingival fibroblasts were grown on culture dishes prior to HAc exposure, no major inflammatory response was observed, and there were no changes in the expression of MMPs [38,67]. Perhaps the culture conditions—seeding cells on HAc-coated plates—versus seeding cells and then exposing them to HAc caused a different response [39,40]. We, therefore, have to be careful when comparing studies that follow a similar but not the same experimental strategy. It would be worth evaluating the impact of the culture conditions on the cell response to HAc in a future study by RNAseq.

Our RNAseq approach has identified a series of genes that are differentially expressed in gingival fibroblasts exposed to HAc—however, the interpretation of these observations is complex. First, the expression changes caused by HAc are relatively low, with only 12 genes reaching the threshold 1.5 log2 change and *p* < 0.1 compared to the >7.0 log2 change of numerous inflammatory genes in the same setting with PRF lysates [42]. Our RT-PCR analysis confirmed the reproducibility of the gene panel identified in the Volcano plot. Thus, there is a restricted selection of genes for setting up a bioassay that reflects the HAc response to gingival fibroblasts and perhaps other mesenchymal cells. Even though gene ontology identified a possible role of apoptosis to be caused by HAc, neither we nor others could identify a possible role of HAc in fibroblast viability [38]. We can only speculate about HAc’s possible impact on the regulated genes’ function. It is also futile to speculate about the clinical meaning of the in vitro findings, particularly because the changes in the transcriptome are moderate to low. There is consequently no obvious gene enrichment that would point toward a biological response. Perhaps the most reliable interpretation is a reversed view—namely that HAc is not a major driver of catabolic events potentially caused by fibroblasts, as we experience them in periodontitis [69] and periimplantitis [70,71]. Clear insights will require a similar single-cell transcriptomic profiling of oral tissues from patients upon HAc application.

At the moment, we can interpret single genes regulated by HAc with respect to their function. Of potential interest are, perhaps, PLK3 acidophilic kinases, a family of five members in mammals with a central role in cell cycle and related events [72], and SLC16a6 involved in ketone body excretion [73,74]. FOXC2 null mice show axial skeletal anomalies caused by impaired hypertrophic chondrocyte remodeling [75]. HB-EGF triggers mesenchymal cell proliferation and differentiation and needs to be tightly controlled to maintain bone and articular cartilage integrity [76]. DGKE protects against renal ischemia/reperfusion injury in mice [53], and its loss causes cell stress [77,78]. DUSP4 and DUSP6 are linked to MAPK signaling in cancers [79], and in our analysis, DUS4 and DUSP6 both appear on the Volcano and heat map, respectively. ATAD2B belongs to the bromodomain proteins and functions as epigenetic readers that recognize acetylated histone tails [80]. PLXNA2 mediates axon guidance in neural development but is also related to osteogenic factor BMP2 [81]. PTGS2 is a key enzyme for prostaglandin E2 synthesis, and it not only drives inflammation but is also relevant to bone homeostasis and regeneration [82,83]. Even though most of the genes can be linked to inflammation and bone biology, the changes in the genetic signatures of gingival fibroblasts only provide one piece of a puzzle to understand the clinical impact of our HAc preparation at a cellular level.

Our study has further limitations; for instance, the in vitro bioassays do not reflect the in vivo clinical situation where the concentration of supplementary HAc is approximately five times higher than we have used in vitro. Even though the dilution now allows pipetting the HAc, it cannot be ruled out that the observed effects are secondary to the high concentration of HAc. A primary effect of HAc needs low concentrations, considering the possible CD44 receptor signaling by the fibroblasts. Future research should, therefore, consider a dose-response of major target genes—for instance, PLK3, SLC16A6, HBEGF, DGKE, DUSP4, PTGS2, and FOXC2—working with even higher dilutions of HAc but still keeping enough HAc in the system to potentially activate CD44 signaling. Indirect support for the activation of CD44 signaling by HAc in oral fibroblasts comes from the activation of ERK and PI3K/AKT-dependent pathways [38]. Future research could follow a siRNA approach to lower CD44 signaling [67,84] or implement mesenchymal cells from knockout mice [84] to understand if CD44 mediates the expression changes we have identified by RNAseq. This is not self-evident, as there is also RHAMM [85]. Future research should further consider the impact of cross-linking HAc with butanediol diglycidyl ether on the cell response, as the ratio is 8:1 of cross-linked versus natural HAc in our clinically used preparation. Care should be taken to generalize the findings towards HAc effects; we have exclusively used a commercial preparation with a HAc of around 1,000 kDa, mainly cross-linked, that is used as a space filler, while HAc of 20–200 kDa takes part in biological processes such as embryonic development and wound healing [86,87]. Another option for screening is to study other cell types, such as macrophages; for instance, however, murine RAW 264.7 cells showed no inflammatory response when exposed to highly purified pharmacological-grade hyaluronan of different molecular weights [88]; we also observed no inflammatory response with HAc. Thus, HAc is not an obvious driver of cytokine or chemokine expression in a setting that is highly sensitive to expression changes.

## 5. Conclusions

We conclude that gingival fibroblasts show a modest response when exposed to HAc, considering that only a few genes display considerable expression changes. Based on this expression pattern, we identified a panel of genes that may serve as a bioassay for further studies on the HAc response of fibroblasts and other mesenchymal cells. In general, the moderate changes in the genetic signature of gingival fibroblasts point toward a rather neutral cell response to HAc in vitro. Considering that fibroblasts produce HAc, the findings of a weak response to HAc are not particularly surprising. Further research should refine our knowledge of how fibroblasts—but also epithelial and other oral cells—respond to HAc and reveal the impact of molecular weight on cross-linking even further, also asking for the underlying signaling pathways.

## Data Availability

All data are available on demand.

## Supplementary Materials

The following supporting information can be downloaded at https://www.mdpi.com/article/10.3390/bioengineering11121307/s1, File S1: Gene regulation lists; File S2: PCA analysis, File S3: RT-PCR; File S4: Heat map; File S5: STRING analysis; File S6: g:Profiler analysis.
