# Supplementary figures and images for: RNA Sequencing Revealed a Weak Response of Gingival Fibroblasts Exposed to Hyaluronic Acid
Source: Bioengineering (Basel). 2024 Dec 23;11(12):1307. doi: 10.3390/bioengineering11121307 (PMC11726844; doi:10.3390/bioengineering11121307)

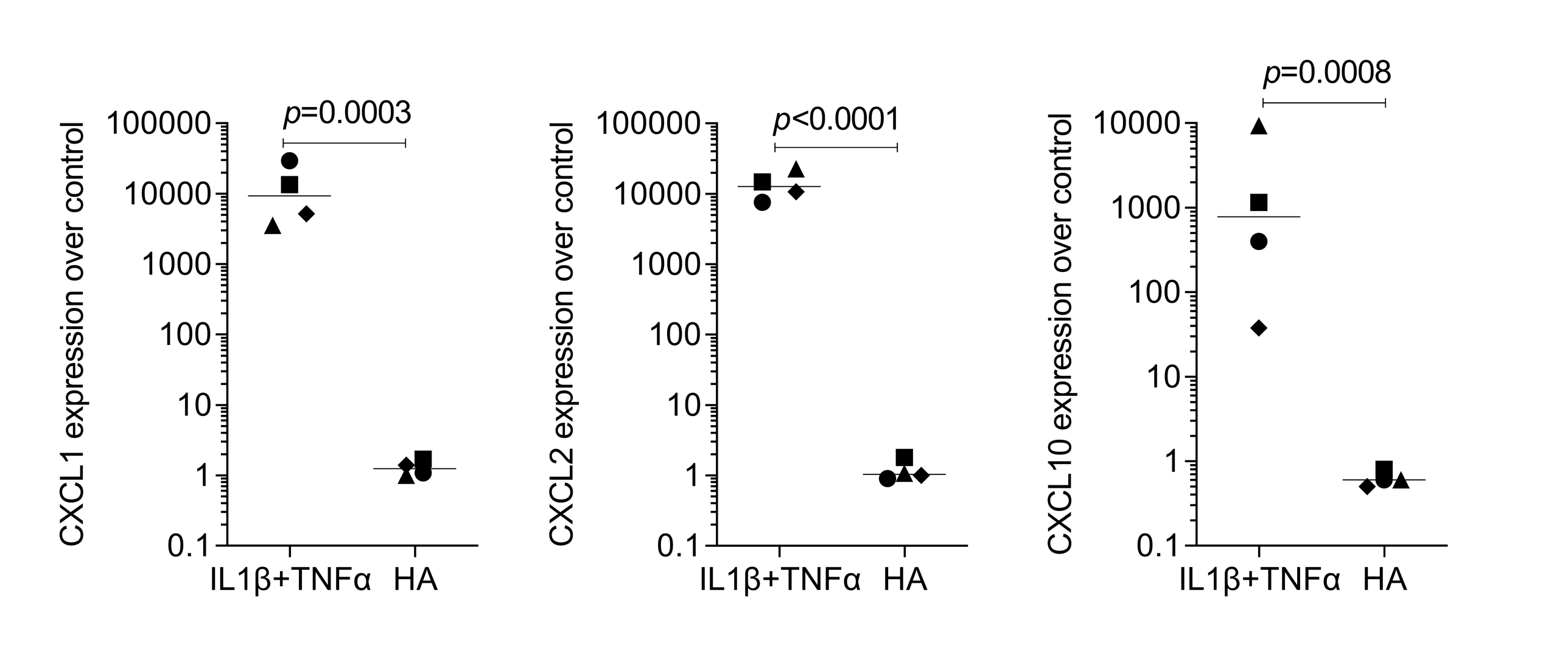

Supplement: Supplementary file 1 [file bioengineering-11-01307-s001.zip › 3. RT-PCR/Figure 1_GF.tif]

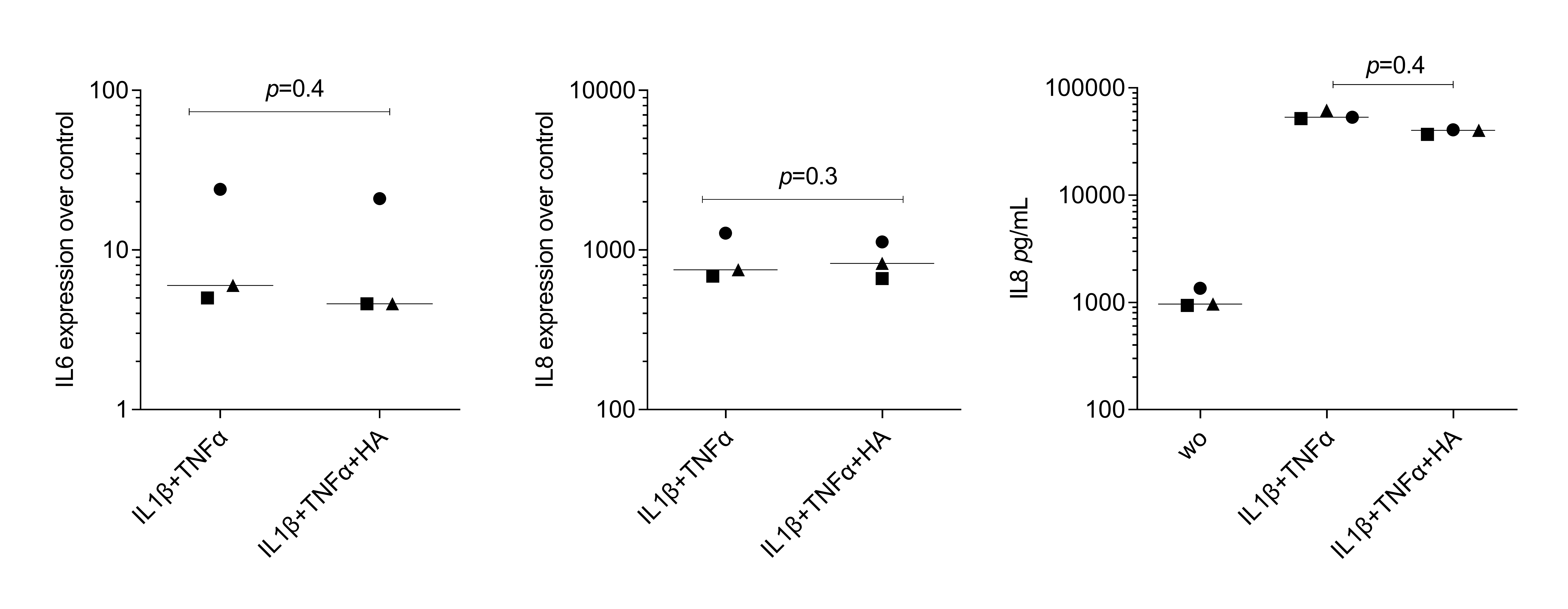

Supplement: Supplementary file 1 [file bioengineering-11-01307-s001.zip › 3. RT-PCR/Figure 2_GF.tif]

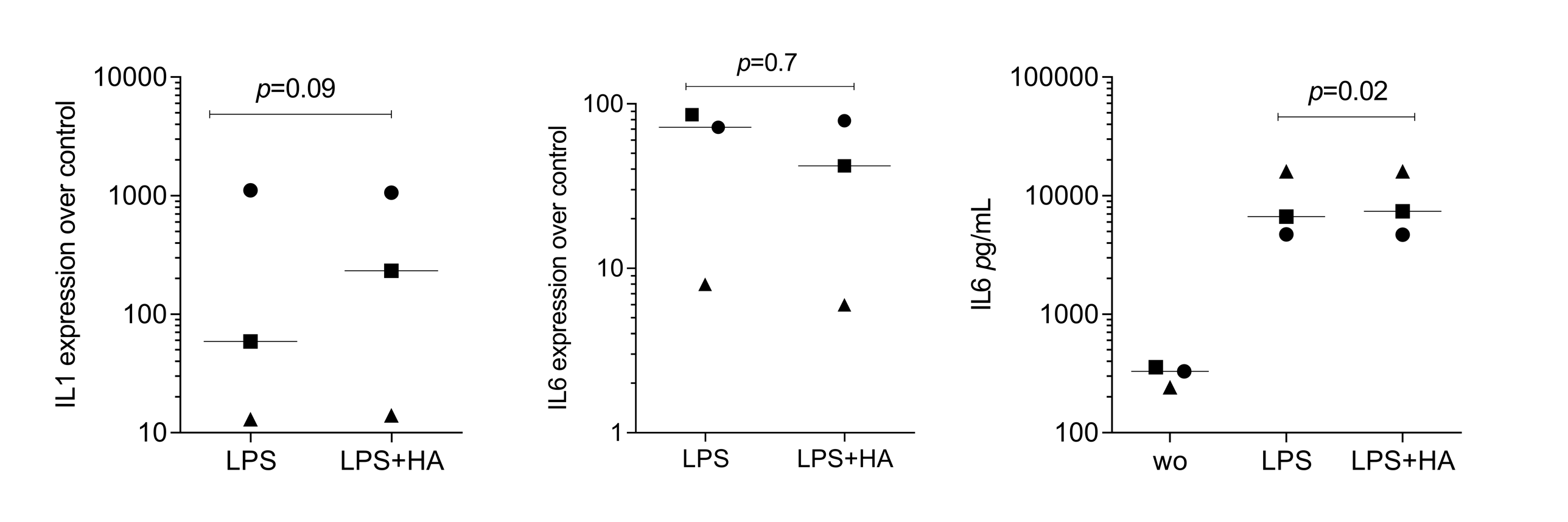

Supplement: Supplementary file 1 [file bioengineering-11-01307-s001.zip › 3. RT-PCR/Figure 3_Macrophages.tif]

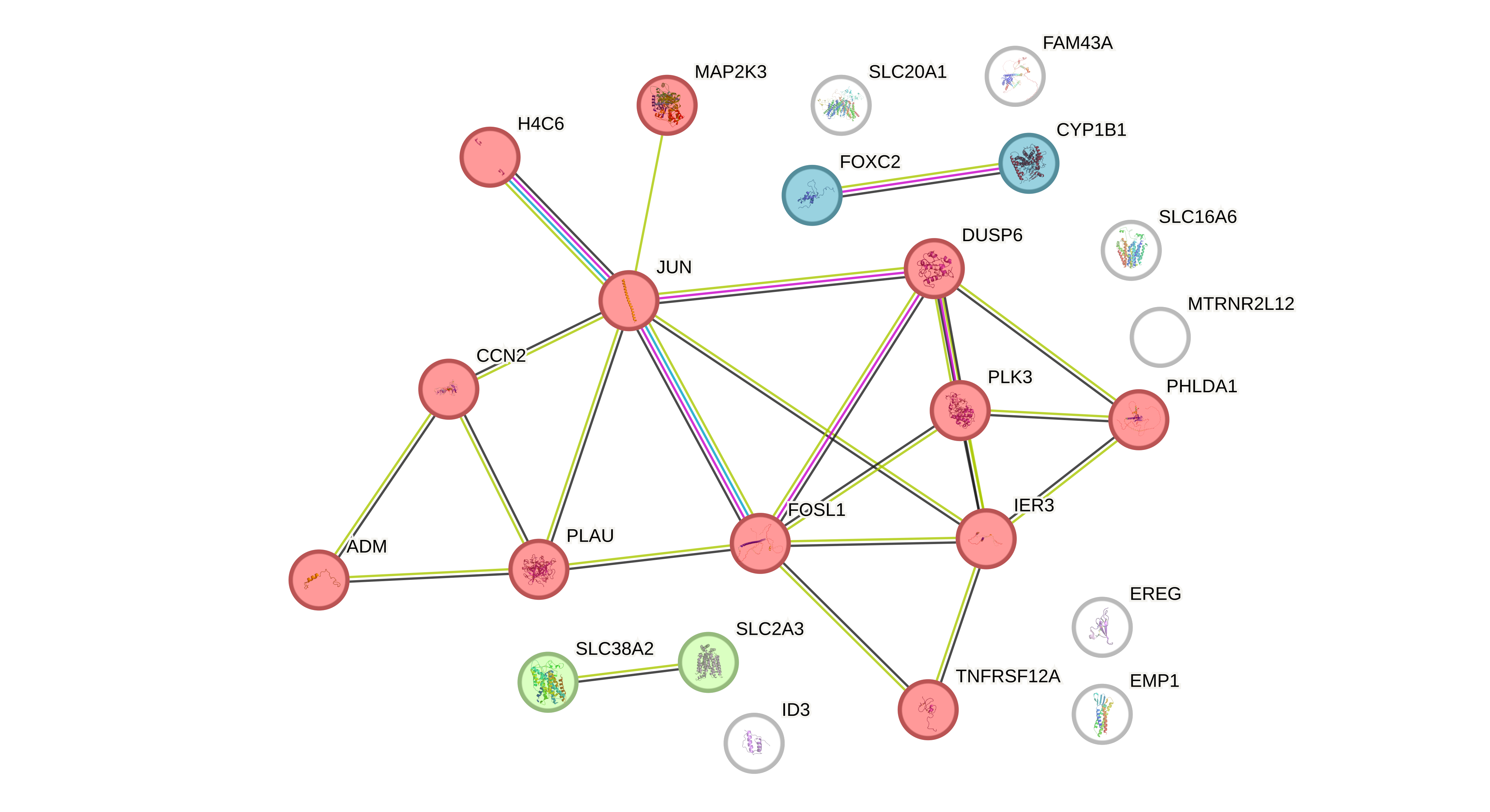

Supplement: Supplementary file 1 [file bioengineering-11-01307-s001.zip › 5. STRING based on Heat Map/string_hires_image.png]

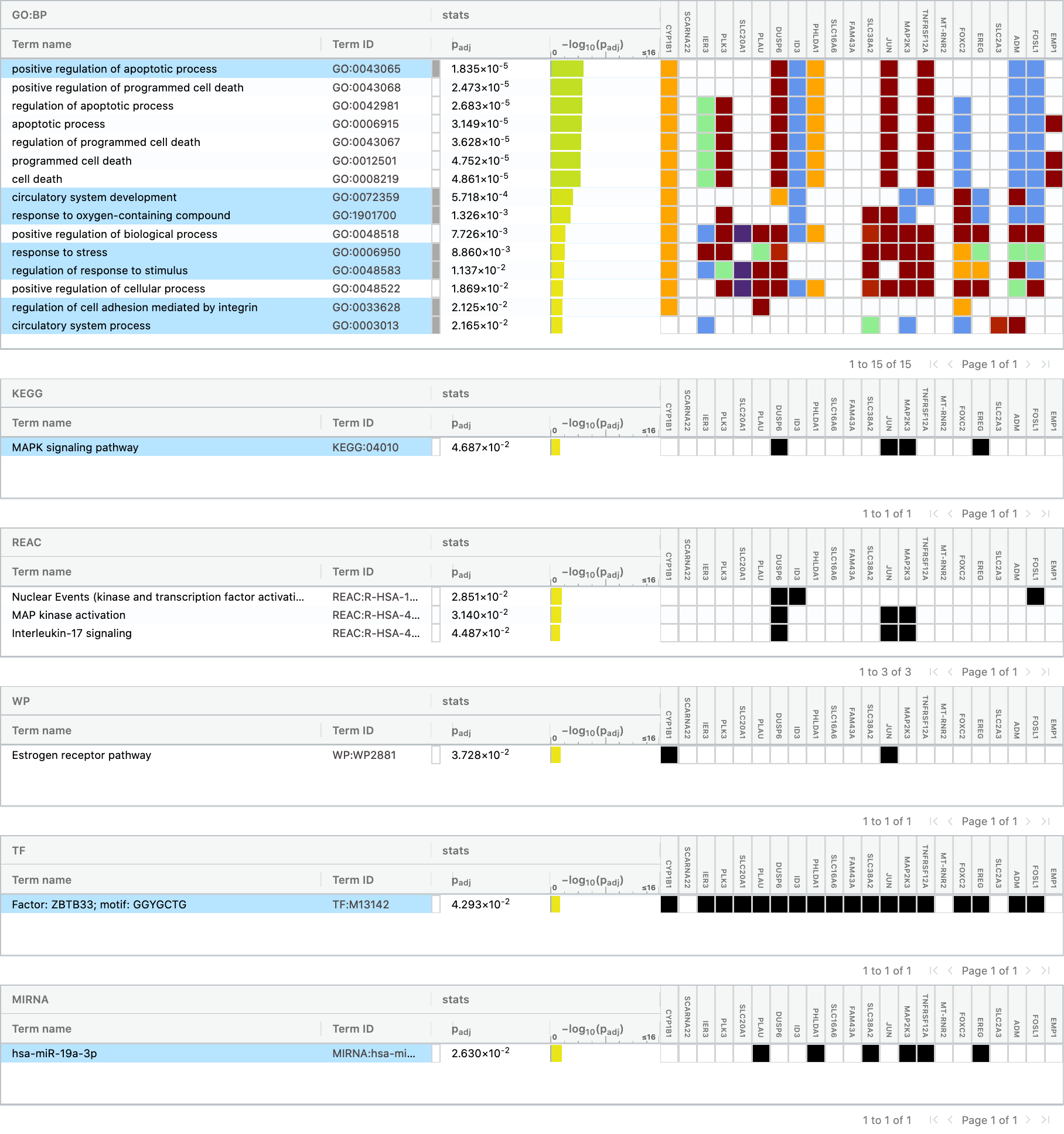

Supplement: Supplementary file 1 [file bioengineering-11-01307-s001.zip › 6. gProfiler based on Heat Map/gProfiler_1.png]

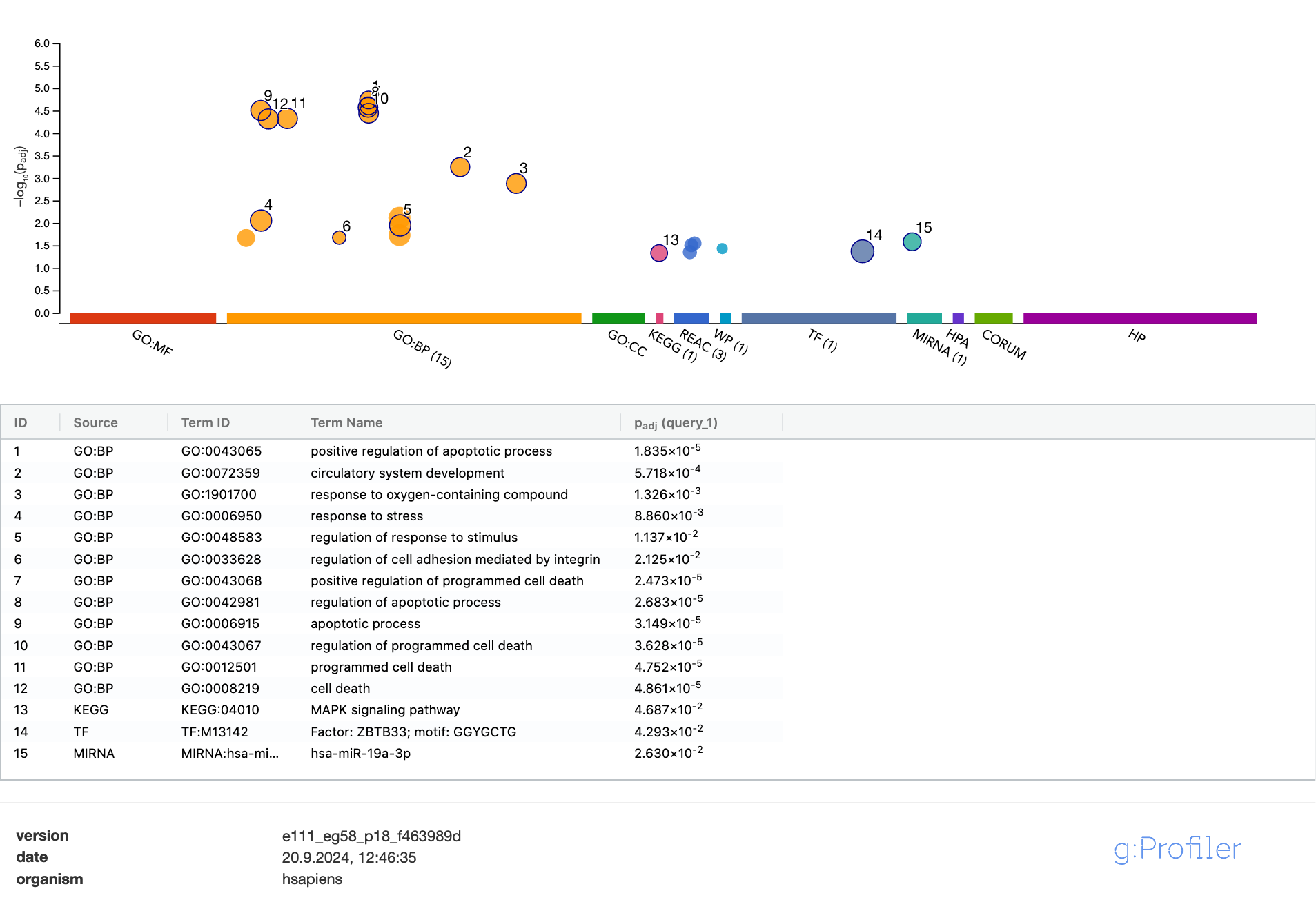

Supplement: Supplementary file 1 [file bioengineering-11-01307-s001.zip › 6. gProfiler based on Heat Map/gProfiler_2.png]
